# Correction: In-person social isolation in the age of smartphones: examining age, period, cohort effects by gender

**DOI:** 10.1371/journal.pone.0340147

**Published:** 2026-01-02

**Authors:** The PLOS One Staff

## Notice of Republication

This article was republished on December 17, 2025. The word “Smartphones” is misspelled in the article title. The correct title is: In-person social isolation in the age of smartphones: Examining age, period, cohort effects by gender. The correct citation is: Peng S, Bauldry S (2025) In-person social isolation in the age of smartphones: Examining age, period, cohort effects by gender. PLoS One 20(9): e0333493. https://doi.org/10.1371/journal.pone.0333493

## Supporting information

S1 File Originally published, uncorrected article. (PDF)

S2 File Republished, corrected article. (PDF)
