# Supplementary material for: In-person social isolation in the age of smartphones: Examining age, period, cohort effects by gender
Source: PLoS One. 2025 Sep 29;20(9):e0333493. doi: 10.1371/journal.pone.0333493 (PMC12478886; doi:10.1371/journal.pone.0333493)
Supplement: S3 Fig — (DOCX) [file pone.0333493.s003.docx]

**
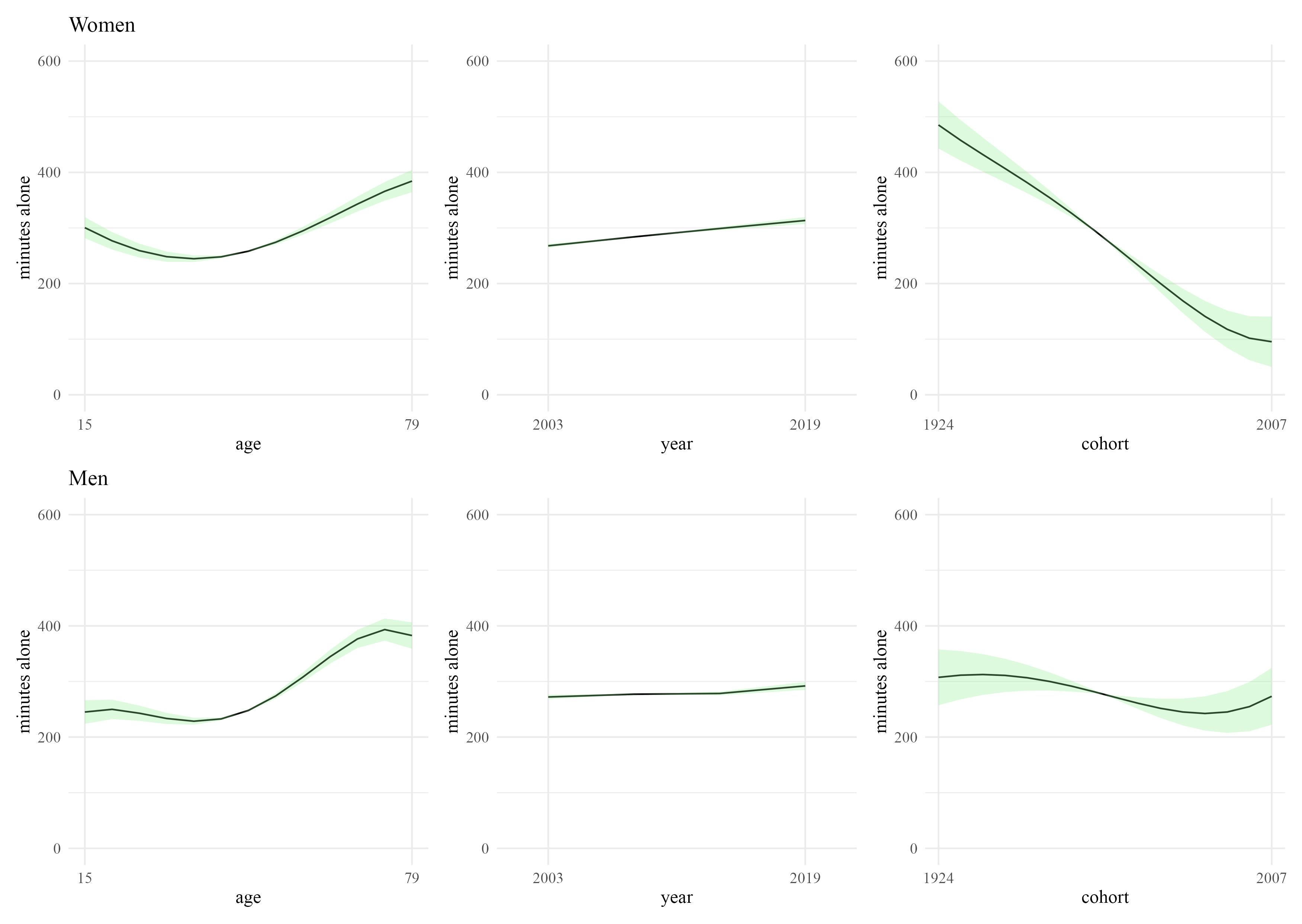
**

**Figure S3. APC model estimates of mean minutes spent alone in nonwork activities by age, period, and cohort for women and men in the pre-pandemic period (2003-2019).** Estimates combine linear and nonlinear estimates from a regression model based on applying the ATUS 2003-22 sample weights and adjusting for holidays and days of the week. Linear estimates reflect the range of effects consistent with the bounded regions of the canonical solution lines given in Figure 2. The black lines represent the midpoint of the bounded linear effects. The green shading reflects the range of possible net total age, period, and cohort effects consistent with the bounded regions of the canonical solution lines.
