# Supplementary material for: In-person social isolation in the age of smartphones: Examining age, period, cohort effects by gender
Source: PLoS One. 2025 Sep 29;20(9):e0333493. doi: 10.1371/journal.pone.0333493 (PMC12478886; doi:10.1371/journal.pone.0333493)
Supplement: Attachment — Submitted filename: isoapc-response-R1.docx [file pone.0333493.s005.docx]

Manuscript ID PONE-D-25-31294 Response memo

| **Editor**  Thank you for submitting your manuscript to PLOS ONE. After careful consideration, we feel that it has merit but does not fully meet PLOS ONE’s publication criteria as it currently stands. Therefore, we invite you to submit a revised version of the manuscript that addresses the points raised during the review process.    The specific comments by the reviewers are reported below. After my evaluation of the manuscript I strongly encourage you to  address in particular the first 2 major points by reviewer 1 (1.1 and 1.2) and all the rather minor recommendations of reviewer 2. Reviewer 1 has also made several good minor points that would improve your manuscript. | |
| --- | --- |
| Thank you for the opportunity to revise and resubmit this paper. We greatly appreciate the valuable feedback provided by the editor and reviewers, which has helped us improve the manuscript. We have carefully addressed all the comments, as detailed in the table below. The left-hand column lists the reviewers' comments, and the right-hand column provides our corresponding responses. All changes in the manuscript were highlighted in yellow. | |
| **Reviewer 1:** | |
| Major Comments and Suggestions  1.1 I recommend extending the analysis to consider gender differences. The literature shows that trends in time-use activities vary significantly by gender, which may influence the period effect. Additionally, time-use patterns by age vary depending on the day of the week. I suggest conducting separate analyses for weekdays (Monday–Friday) and weekends/holidays (Saturday, Sunday, and public holidays). Discretionary time (and potentially time spent alone) is distributed very differently across these types of days, and it would be interesting to explore this heterogeneity. | Replaced primary analysis with analysis stratified by gender. Please see updated Figures 1-3. We also revised the texts throughout the paper to reflect this change of analysis by gender.  We compared weekday and weekend patterns and included total effect estimates in the supplementary materials (Figure S1). Overall trends are similar across both, but magnitudes differ: the age effect is more pronounced on weekdays, while period and cohort effects are more pronounced on weekends. Although these differences are interesting, the further stratifying of results and figures by weekend and weekday may confuse readers. Therefore, we presented this in the supplemental materials and added the following paragraph to the Sensitivity Analyses to introduce this new analysis:  “Although our main analysis adjusts for day of the week and holidays, we also conducted a stratified analysis by weekday versus weekend to explore potential differences. As shown in Figure S1 (supplementary material), overall effects are similar across both, but magnitudes differ: the age effect is more pronounced on weekdays, whereas period and cohort effects are more pronounced on weekends.” (p. 13) |
| 1.2 Could the method and analysis be applied specifically to free or discretionary time spent alone? This would help isolate individual choice from more structured or obligatory time-use patterns (e.g., education). It could also strengthen the argument that societal shifts are driven by the rise of smartphones and social media, helping to control for other factors such as increased online learning post-pandemic. | As suggested, we conducted an analysis using leisure time spent alone (see Figure S2 in the supplementary material). The results are largely consistent with our main analyses, though the cohort effect for women is attenuated—from a threefold to a twofold difference—indicating that cohort differences in alone time are larger for non-work time than for leisure time. Overall, age, period, and cohort effects are similar in both contexts. Therefore, we retained non-work time as our primary measure to align with the Surgeon General’s report and other work in this area. We added the following paragraph to the Sensitivity Analyses to introduce this new analysis:  “Rather than focusing on nonwork alone time, there are alternatives ways to operationalize free or discretionary time, such as leisure time. Leisure time is measured as activities such as Eating & Drinking (activity code 110101 – 119999 and 050202), Socializing, Relaxing, & Leisure (activity code 120101 – 129999 and 050201), and Sports, Exercise, & Recreation (activity code 130101 – 139999 and 050203). We conduct sensitivity analysis using leisure time spent alone (see Figure S2 in the supplementary material). The results are largely consistent with our main analyses, though the cohort effect for women is attenuated—from a threefold to a twofold difference—indicating that cohort differences in alone time are larger for non-work time than for leisure time. Overall, age, period, and cohort effects are similar in both contexts, suggesting the robustness of our findings. Therefore, we retain non-work time as our primary measure to align with the Surgeon General’s report and other work in this area (1,10).” (p.13) |
| 1.3 How sensitive are your findings to the COVID period? Time spent alone changed substantially during the pandemic. Please also refer to the comment below regarding the use of sample weights during this period. | Our estimates of total effects are broadly similar when we conduct the analysis just using pre-pandemic data (2003-2019). The cohort estimates are slightly stronger for women and weaker for men than in the primary analysis. Clearly the pandemic had some effect, but it did not alter the general pattern of age, period, and cohort effects for men and women. We include a figure of total effects in the supplementary material (see Figure S3). We added following paragraph in the sensitivity analysis:  “Given the COVID-19 pandemic has influenced people’s social interactions (4), we conduct sensitivity analysis to examine whether our main findings are robust using pre-pandemic data (2003-2019). Our estimates of total effects are broadly similar when we conduct the analysis just using pre-pandemic data (2003-2019). The cohort estimates are slightly stronger for women and weaker for men than in the primary analysis. Clearly the pandemic had some effect, but it did not alter the general pattern of age, period, and cohort effects for men and women. We include a figure of total effects in the supplementary material (see Figure S3).” (p. 13-14) |
| Minor Comments 1. In the abstract, the authors describe a 10% increase in the period trend over 20 years as "modest." While it may be modest relative to age and cohort effects, it is not necessarily modest in absolute terms and could be rephrased for clarity. | We removed the word “modest” as suggested. We also revised the results section of the abstract to reflect our new analysis:  “Social isolation has increased over the past two decades for both women and men, with a notable acceleration in the mid-2010s, suggesting that societal shifts during this period may have intensified isolation. However, age and cohort effects play a much larger role in explaining the social isolation crisis of the 21st century. Gender differences are substantial, particularly in cohort trajectories and in age patterns after age 65.” (p. 2) |
| 2. The presentation of the time-use data is appreciated, particularly for readers familiar with the dataset. However, more background information would benefit general readers who are less familiar with the data. | We revised and added more background information about ATUS and time diary data in the METHOD section:  “Data for this study come from the 2003 through 2022 waves of the American Time Use Survey (ATUS), which is conducted by the U.S. Census Bureau with funding from the Bureau of Labor Statistics to collect information on how Americans spend their time. The ATUS is a nationally representative sample that draws on a subset of households from the Current Population Survey (CPS) each month. An eligible person from the household (a person of at least 15 years) was randomly selected to be interviewed. Interviews were conducted by telephone using a computer-assisted telephone instrument (CATI). The time diary provided a 24-hour recall from each respondent, starting at 4:00 a.m. the previous day and ending at 4:00 a.m. on the interview day. Interviews usually took place the day after the reference day to ensure the events were still fresh in memory. ATUS collected information about what the respondent was doing, how long each activity lasted, where each activity occurred, and whom the respondent was with. The shortest unit of time reported for a given activity is 5 minutes, which allowed for up to 288 activities on a given day, thus providing a finely-grained portrait of everyday social dynamics. The analysis sample consists of respondents between the ages of 15 and 79, which over the 20-year period results in an N of 240,576 cases.” (p. 4-5) |
| 3. The ATUS definition of "time spent alone" introduces some ambiguity. The definition states: “Time spent alone includes activities in which ATUS respondents were asked who else was with them during an activity but did not report that others were present.” It is unclear whether “present” means physically present. For example, during online gaming or social media interactions, respondents may be physically alone (e.g., in a room) but socially engaged with others. If respondents report they were "with others" in such cases, the measure of physical solitude may be understated, potentially leading to an underestimation of the findings. | The ATUS “Who” question captures only individuals physically present with the respondent in the same location. The User’s Guide states: “TUWHO_CODE (Information about who was in the room with the respondent.”)  <https://www.atusdata.org/atus/resources/linked_docs/atususersguide.pdf>  We verified this interpretation by cross-checking digital interaction activities (codes 160101–169999) in the “What” question and found that these episodes are coded as alone under the “Who” variable.  This study focuses on in-person social isolation, as reflected in the title—“In-person social isolation in the age of smartphones: examining age, period, cohort effects by gender”. This focus is warranted because our goal is to understand how the smartphone era has influenced face-to-face social isolation. To ensure clarity, we explicitly describe this in the Measures section:  “The ATUS collects “Who” questions for all activities, except for sleeping and personal activities (e.g. grooming, cuddling, having sex, etc.). “Who” questions are used to determine whether respondents spent face-to-face time with others or alone.” (p. 5) |
| 4. In the same vein, excluding time spent alone while working (particularly from home) could further understate the results, especially given the increasing prevalence of remote work since the pandemic. | As noted by Reviewer 1 (Comment 1.2), distinguishing between discretionary time spent alone and obligatory activities (e.g., work) is important, as these forms of alone time may be shaped by different factors. Therefore, we focus on nonwork time as our primary measure, consistent with the Surgeon General’s report and prior research in this area (1,10). |
| 5. How did you handle sample weights for 2020 (wt20), which are incompatible with the weights from other years (wt06)? Please clarify how you combined or adjusted them in the analysis. | We followed ATUS guidance (<https://www.bls.gov/tus/atususersguide.pdf>) on weighting for each year. Specifically, for survey year 2003–2019 and 2021–2022, we applied the final ATUS person weights (wt06)), and for 2020, we used the pandemic-adjusted weights (wt20). These weights were applied as provided by ATUS without further adjustment because our analyses focus on regression modeling and relative differences rather than pooled population totals. Using the appropriate weight for each year preserves representativeness within that year and aligns with ATUS recommendations for analyses spanning this period.  As noted by Reviewer 1 (Comment 1.3), an alternative approach to address the survey weight issue is to exclude 2020 data from the analysis. We conducted a sensitivity analysis using only pre-pandemic data (2003–2019) and found that the main findings remained robust, indicating that our results are not driven by the inclusion of pandemic data or its associated survey weights. |
| 6. The method for constructing cohort groups is not clearly described. Please provide more detail, similar to the explanation given for age and period groups. | We added following details to the measurement:  “Finally, we took the difference between the period and age indicators, which resulted in 16 eight-year cohort groups beginning with the 1924 birth cohort, the oldest in the sample, and ending with the 2007 cohort, the youngest in the sample (i.e., 1924-1932, …, 1999-2007).” (p. 6) |
| 7. In Equation (1), the subscripts L and NL presumably refer to linear and nonlinear components, respectively. Please clarify this explicitly in the text. | Revised as suggested on p. 7:  “the subscript *L* refers to linear components, while the subscript *NL* refers to nonlinear components.” |
| 8. On page 7, change the reference “from (4)” to “from Equation (4)” to avoid confusion with citation references. | Revised as suggested. |
| 9. The use of the term “unconditional” to describe the results is ambiguous. Does it refer to simple averages or raw values without covariate adjustment? Please define the term more precisely. | This is a good point. We removed the term “unconditional” on p. 10 in reference to Figure 1 and instead referenced that Figure 1 illustrates simple averages. |
| 10. Figure 2 is difficult to interpret. Consider adding numerical labels to the axes to improve readability. For example, the linear period effect of 0, linear age effect of 4.7, and cohort effect of 0.2 are mentioned in the text but are not clearly indicated in the figure. The same applies to the midpoint; please label these values directly in the graph to guide the reader. | We tried adding three coordinates for each line to the figure, but it was too much text. Instead, we reference specific values in the text and in the caption for the figure. |
| **Reviewer 2:** This study examines the temporal effects of social isolation in the United States. Using data from the American Time Use Survey from 2003 to 2022, the authors employ an Age-Period-Cohort (APC) model to distinguish between the impacts of getting older, changes in society over time, and generational effects. Overall, this is a generally well-executed paper that could be strengthened with a few minor revisions. | |
| 2.1. In the "Analytic Method" section, the paper should more explicitly state its significant reliance on the work of Fosse and Winship. This is in terms of notation, model set up, approach, visualization, etc. | As suggested, we added following sentences in the "Analytic Method" section in p. 6:  “The current state of the art for APC analyses, as proposed by Fosse and Winship (9,22),”  “In this study, we applied the Fosse and Winship APC approach (9,22) to address our research questions. We adopt their notation in describing the models and recommendations for illustrating results.” |
| 2.2. For greater clarity and consistency with the cited Fosse and Winship methodology, the authors should consistently use the term "effects" rather than using it interchangeably with "trends." In the context of an APC analysis, "effects" refer to the influence of the distinct underlying variables that age, period, and cohort represent, whereas "trends" are more appropriately used to describe the observable patterns in the data over time. In short, strike out the use of the word "trends" in the paper as this is an APC analysis. This wouldn't take much time at all and would greatly increase the clarity and scope of the paper. | “Trends” are replaced with “Effects” across the paper. |
| 2.3. The conclusion would be improved by adding a paragraph that emphasizes the tentative nature of the findings. An APC analysis is based on a rank deficient design matrix, meaning there are infinite possible solutions that fit the data; the selection of a particular solution is guided by theoretical assumptions rather than being solely data-driven, as the authors are no doubt aware. | We included a paragraph in the conclusion emphasizing this point for readers:  “It is important to keep in mind that disentangling age, period, and cohort effects is not possible empirically due to the linear relationship between the three components that results in a rank deficient design matrix. An infinite number of potential combinations of age, period, and cohort effects are consistent with any given data and, ultimately, it requires theoretical assumptions and substantive knowledge to impose restrictions to help eliminate some of the infinite possibilities. We have imposed what we believe are modest assumptions, but this is also an avenue for future studies—identifying stronger or alternative assumptions that can further narrow the bounds on age, period, and cohort effects in time spent alone.” (p. 15) |
